# *Wnt *Signaling: A Potential Therapeutic Target in Head and Neck Squamous Cell Carcinoma

**DOI:** 10.31557/APJCP.2019.20.4.995

**Published:** 2019

**Authors:** Zeeshan Javed, Hafiz Muhammad Farooq, Mukhtar Ullah, Muhammad Zaheer Iqbal, Qamar Raza, Haleema Sadia, Raffaele Pezzani, Bahare Salehi, Javad Sharifi-Rad, William C Cho

**Affiliations:** 1 *Center for Applied Molecular Biology,*; 3 *Center for Excellence in Molecular Biology, University of The Punjab, Lahore, *; 2 *Institute of Biochemistry and Biotechnology, University of Veterinary and Animal Sciences, Pakistan, *; 4 *OU Endocrinology, Department of Medicine (DIMED), University of Padova, via Ospedale 105, *; 5 *AIROB, Associazione Italiana per la Ricerca Oncologica di Base, Padova, Italy, *; 6 *Student Research Committee, School of Medicine, Bam University of Medical Sciences, Bam, *; 7 *Food Safety Research Center (salt), Semnan University of Medical Sciences, Semnan, Iran,*; 8 *Department of Clinical Oncology, Queen Elizabeth Hospital, Hong Kong SAR, China. *

**Keywords:** Wnt signaling, therapeutic target, head and neck squamous cell carcinoma

## Abstract

Cellular maintenance and development are two fundamental mechanisms regulated by the canonical *Wnt* signaling pathway. *Wnt*/*beta-catenin* signaling pathway controls a myriad of cellular processes that are essential for normal cell functioning. Cell cycle progression, differentiation, fate determination, and migration are generally orchestrated by canonical *Wnt* signaling. Altered *Wnt/beta-catenin* signaling has been considered a promoting event for different types of cancers and the oncogenic potential of *Wnt* signaling have been discussed in many cancer types, including breast, colon, pancreatic as well as head and neck. Furthermore, *Wnt* signaling is critical for the maintenance and stemness of both the normal as well as cancer stem cells. This review sheds new light on *Wnt* signaling and explains how it can regulate normal physiological processes and curtail the development of cancer. It depicts the vital functions of *Wnt* signaling in the stem cell growth and differentiation by focusing on current druggable targets that have been ascribed by recent studies. Thus, *Wnt* signaling pathway retains a tremendous potential in eradicating head and neck squamous cell carcinoma.

## Introduction

Head and neck squamous cell carcinoma (HNSCC) are one of the most aggressive cancer types with high fatality rate due to late diagnosis and high recurrence. It arises from the mucosal surfaces of various organs with epithelial linings. Common organs include sinuses, oral cavity, tongue, pharynx, and larynx (Sengupta, 2012). The incidence rate of HNSCC overall in the United States is 11 per 100,000 individuals and is more prevalent in black population compared to white people, and it is the most prevalent type of tumor after cervical cancer (Fakhry et al., 2018). Head and neck squamous cell carcinoma accounts for more than 330,000 death worldwide with more than 650,000 cases of HNSCCs reported annually (Bray et al., 2018). In the US alone it has been reported that HNSCCs was responsible for 10,800 deaths and more than 53,000 American population developed the disease on an annual basis (Bray et al., 2018; Siegel and Miller, 2019). In addition to this HNSCCs incidence comprises 3% of all malignancies in the US and 4% of all cancer-related malignancies in Europe respectively. However, in Europe, nearly 250,000 cases of HNSSCs are recorded each year, and death tally is 63,500 per year. HNSCCs is more prevalent in males as compared to females with ratios between 2:1 to 4:1 respectively. The incidence rate of HNSCCs is 20 per 100,000 in different regions of the world moreover, HNSCCs is most common type cancer In Asian countries (Bray et al., 2013) Risk of recurrence, unavailability of suitable therapies and low efficacy of currently used drugs are the major setbacks that have hampered the efforts towards a valid therapeutic approach for the treatment of HNSCC (Haddad and Shin, 2008; Javed et al., 2016).

Like any other tumor, it has become really difficult to treat HNSCCs. Overall it is the sixth most prevalent type of cancer with survival rate less than 60% (Bae et al., 2016), like other cancers, physicians can hardly treat HNSCC patients as they are facing several hindrances such as the rapid growth of tumor, in availability of early medical care, reluctance of people towards early treatment and the presence of cancer stem cells (CSC) worsen the tumor progression . Indeed these are a sub-population of cells which are resistant to chemo and radiotherapy and frequently promote recurrence by halting or escaping clinical efforts also in HNSCC (Mannelli and Gallo, 2012). CSC show coherent features, i.e. similar to normal stem cells such as self-renewal, development and differentiation properties. Understanding the mechanisms involved in the maintenance of CSC holds promising benefits for delineating the causes of tumorigenesis, metastasis, self-renewal (Mannelli and Gallo, 2012; Routray and Mohanty, 2014). Moreover, understanding cell-cell interaction can aid in the development of appropriate druggable targets for HNSCC and consequently potential treatment. Aberrant *Wnt* signaling pathway has been reported to be a Promoting event for different types cancers , such as colon, breast and lung and even reported to implicate decisive role in safeguarding the CSC, as well as support the fundamental machinery that promotes HNSCC progression (Vermeulen et al., 2010; Lamb et al., 2013; Zhang et al., 2015). This review aims to discuss the involvement of *Wnt* signaling in maintenance of stemness in CSC with particular reference to HNSCC and different therapeutic and diagnostic options for HNSCCs furthermore it shed light on the use of a natural compound as a promising therapeutic agent for the HNSCC treatment. 

*Cancer Stem Cells in HNSCC*

The ability of self-renewal and differentiation are the two main features of stem cells (SC). Stem cells only divide to give rise to the new progeny of cells as they lack any tissue-specific structure (Reya et al., 2001). Depending upon specific cellular signaling SC can undergo cell division to produce either cells with retained pluripotency or progenitor cells for the synthesis of more mature and functionally active cells which can differentiate into more specific cell or tissue. In the definition of SC also falls embryonic stem cells (ESC) which originate from the blastocyst of the embryo. ESC possesses a fundamental ability, pluripotency, a feature that guarantee the development of any tissue or organ. Differently adult stem cells (ASC) are cells characterized as unipotent or multipotent, they reside in the specific tissue and can produce a variety of cells, but not all cells from which the organ was built (Reya et al., 2001; Schepers, 2003; Mannelli and Gallo, 2012).

The origin of CSC came into limelight by the experiments conducted by Bonnet and Dick in 1997 when they successfully transplanted immune-deficient mice with stem-like cells termed as severe combined immunodeficiency disease leukemia-initiating cells (SL-IC). These cells retain the properties of SC in such a way that they can proliferate and differentiate and can produce leukemia. Furthermore, these cells maintain the capacity of self-renewal and have the potential to establish disease in other mice injected with SL-IC (Bonnet and Dick, 1997). Later other studies provided substantial evidence for the existence of CSC in different forms of cancers such as breast, lung, prostate, pancreatic and head and neck (Dick, 1997). Altogether these findings established the existence of CSC and further underlined their ability of pluripotency and self-renewal. Involvement of CSCs in HNSCCs came from Prince and colleagues (Prince et al., 2007). Their findings revealed that expression of CD44+ a lineage marker was indispensable for persistent tumor heterogeneity in HNSCCs. Using limiting dilution assay they confirmed the high prevalence of CSCs in HNSCCs after breast cancer, indicating the fact that cancer stem cells are pivotal for epithelial cells mediated HNSCCs (Prince et al., 2007). CD44+ separated by the aldehyde dehydrogenase (ALDH) has been reported to have more tumorigenic properties and self-renewal capacity compared to normal CD44+ not separated by this enzyme. ALDH has been linked to confer therapy resistance and tumor prevalence via modulating the expression of PI3K (phosphoinositide 3-kinase) and SOX2 (SRY-box 2) signaling cascades. ALDH concentrations have been directly associated with increased tumor size and symptoms of cancer in cell lines of HNSCCs cancer (Bertrand et al., 2014) Apart from CD44 a broad of range of markers have been investigated in CSCs and specifically HNSCCs. CD133 is a transmembrane glycoprotein whose presence have been linked to increase drug resistance against cisplatin in vitro (Silva Galbiatti-Dias et al., 2018). This marker has been studied extensively for its role in modulating the expression of various tumors such as lung cancer, HNSCCs and other solid tumors (Silva Galbiatti-Dias et al., 2018). CD24 another glycoprotein has been reported to hinder the efficacy of drugs like cisplatin gemcitabine by promoting drug resistance in CSCs of HNSCCs (Modur et al., 2016). Several other factors regulates the expression of CSCs in HNSCCs. Enzymes such as ALDH and proteins such as BMI and transcription factors such as the Nanog and SOX2 all have been implicated to play a decisive role in proliferation and drug resistance of CSCs. 

Furthermore, CSC has also been reported to have a longer life span and negate the process of apoptosis due to the presence of drug resistance proteins such as ATP-binding cassettes family transporters. Cancer stem cells trigger tumorigenesis in a variety of cancers such as lung cancer and HNSCCs as they confer drug resistance and disease relapse in these tumors. (Wolmarans et al., 2018; Cojoc et al., 2015).

**Figure 1:**
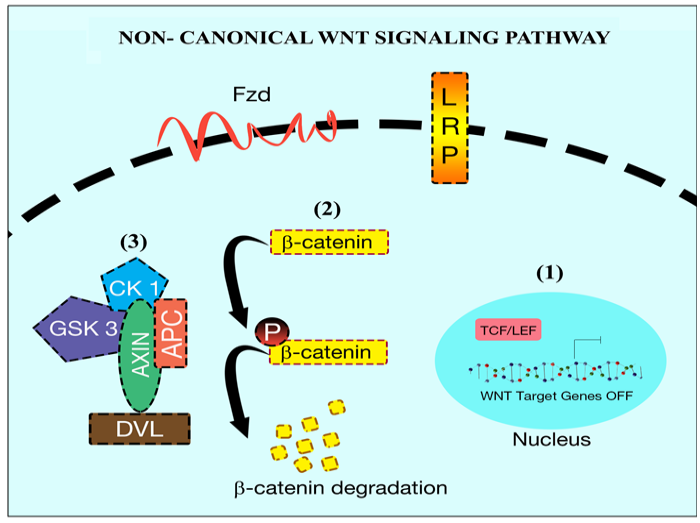
Non-Canonical *Wnt* Signaling Pathway. In the absence of Wnt signaling (1) the β-catenin is targetd for degradation (2) (Phosphorylation) by the complex. The complex consists of tumor suppressor gen (APC), scaffold protein (Axin) and kinase ( CK 1, GSK)(3)

**Figure 2:**
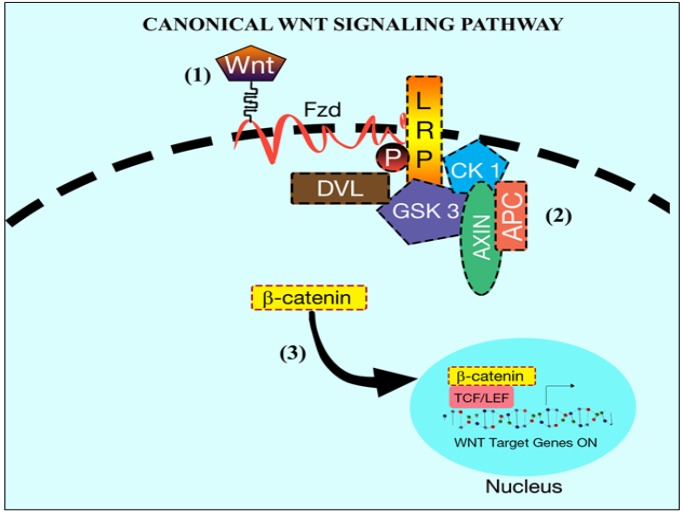
Canonical *Wnt* Signaling Pathway. In the presence of Wnt signaling, β-catenin degradation is inhibited. Wnt binds to Fzd and LRP receptors (1). Cytoplasmic protein DvL facilitates recruitment of the complex (formed by APC, Axin, CK1, GSK 3) at LRP level (2). Consequenlty β-catenin is free to translocate into the nucleus and interact with TCF/LET transcription factors to active target genes (3)

*Wnt Signaling Pathway*

*Wnt* signaling is vital for a plethora of cellular function ranging from homeostasis to the development of mature tissue. Embryonic development also requires *Wnt*-mediated canonical signaling (MacDonald et al., 2009; Takahashi-Yanaga and Kahn, 2010; Novellasdemunt et al., 2015). Moreover, *Wnt* signaling is inevitable for regulating cellular proliferation, apoptosis, metastasis, and migration of cells (Noguti et al., 2012). Of note, *Wnt* operates through either canonical or non-canonical pathways which are differentiated by beta-catenin involvement (Ozbey et al., 2018). 

While the binding of *Wnt* protein initiates the classical *Wnt* signaling to Frizzled transmembrane receptors (Le et al., 2015), non-canonical signaling requires other molecules for downstream activation such as co-receptors Receptor tyrosine Kinase-like orphan receptor 2 (ROR2) or receptor-like tyrosine Kinase (RYK) (Rao and Kühl, 2010). Together these signal transducers activate the calcium-dependent signaling cascade via activation of scaffolding proteins disheveled (DvL). Thus, the initiated signaling aids in the cellular movement and cytoskeleton remodeling by activation of Rho family of GTPases that in turn activates RhoA and Rac. Cellular polarity is mediated by the commencement of c-Jun N-terminal kinase (JNK) signaling cascade through triggered Rac. DvL mediated activation of *Wnt* signaling induces the launch of heterotrimeric G protein which promotes cellular export of calcium to extracellular environment (Katoh and Katoh, 2007; Nalbantoglu et al., 2011). This transportation activates several kinases which include JNK, nemo like Kinase (NLK) and nuclear factor of activated T-cell (NFAT) through activation of calcium responsive kinase C and calcium/calmodulin-dependent Kinase II (CaMKII) (Anastas and Moon, 2013). NFAT via its activation through calcineurin plays a decisive role in the gene expression and embryogenesis (Li and Neaves, 2006; Peerani et al., 2007). On the other hand, non-canonical signaling mediates the activation of canonical *Wnt* signaling through phosphorylation of T-cell factor (TCF) via NLK (Katoh and Katoh, 2007; Rao and Kühl, 2010). This indicates that both canonical and non-canonical signaling are antagonized one each other and calcium modulates such counteract by either activation of one the signaling (Nalbantoglu et al., 2011) (Figure1).

Differently, canonical *Wnt* signaling is commenced by the binding of *Wnt* to the cytoplasmic beta-catenin signaling cascade (Logan and Nusse, 2004). In the absence of *Wnt*, beta-catenin is normally degraded by the E3 ligase. Cytoplasmic beta-catenin usually exists freely or attached to APC, but a trimeric complex composed by Axin, CK1 and GSK3 phosphorylate cytoplasmic beta-catenin at serine residues via phosphorylation of Axin and APC making beta-catenin vulnerable for the E3 ubiquitination and degradation (Figure 1) Non-canonical *Wnt* signaling pathway. In the absence of *Wnt* signaling (1) the *β-catenin *is targeted for degradation (2) (Phosphorylation) by the complex. The complex consists of a tumor suppressor gene (APC), scaffold protein (Axin) and kinase (CK 1, GSK) (3). Activation of Axin-CK1-GSK3 complex initiates the phosphorylation of APC that promotes beta-catenin degradation. When there is the activation of canonical *Wnt* signaling, *Wnt* can attach to Fzd along with lipoprotein receptor-related protein (LRP) to form a trimeric complex (MacDonald et al., 2009). This trimeric complex helps in the phosphorylation of DvL. Phosphorylation of DvL recruits Axin-CK1-GSK3 complex to the cell membrane preventing it from phosphorylating APC which is attached to beta-catenin, thus allowing cytoplasmic beta-catenin accumulation and translocation into the nucleus where it can activate *Wnt* target genes (Ilyas, 2005; Katoh and Katoh, 2007; Wu and Pan, 2010) (Figure 2).

*Wnt* signaling has been implicated in a broad range of cellular processes such as cell growth, differentiation, and migration: at the same time, it inhibits cell death through apoptosis. Transcription and signaling of molecules such as Cyclin D1, c-Myc, cyclooxygenase 2 (COX2), bone morphogenetic protein 4 (BMP4), matrix metalloproteinases7 (MMP7), Axin2, multi-drug resistance 1 (MDR1), b-TRCP and endothelial growth factor (EGF) have been reported to be under the influence of the *Wnt*-mediated canonical signaling, suggesting that *Wnt* has the key authority in sustaining cell development, migration and early embryogenesis (Van Roy and Berx, 2008).

*Wnt Signaling in HNSCC*

HNSCCs involves the spread of malignant cells at multiple sites. It is a malignancy characterized by distinctive heterogeneity between inter and intra-tumor environment (Puram et al., 2017) (Puram et al., 2017). HNSCCs are divided into Human papillomavirus-positive (HPV+) and HPV- tumors. Each type is bearing its characteristic features of clinical, pathological and epidemiological significance (Castilho et al., 2013; Castilho and Gutkind 2014). Abrogated *Wnt/Beta-catenin *signaling has been a hallmark in tumor biology of both HPV+ as well as HPV- types HNSCCs (Nwanze et al., 2015). Many factors play a crucial role in the development of the tumor. Epigenetic modifications in (surfactin exporter in surfactin self-resistance) SRFP, (*Wnt* inhibitory factor) WIF and (dickkopf *WNT* signaling pathway inhibitor 3) DKK3 that inhibit the *Wnt* signaling cascade. However, mutation prompts them to activate the *Wnt* Signaling in the basal cells of HNSCCs (Pannone et al., 2010; Pannone et al., 2010). Overexpression of Beta-catenin has been strictly related to the increased transcriptional activity in HNSCCs (Yu et al., 2018; Hu et al. 2015). Derailed expression of the Beta-catenin can, in turn, promotes poor prognosis and increased differentiation in HNSCCs (Padhi et al., 2015; Padhi et al. 2015). Epigenetic changes that promote growth and regulation of cancerous stem cells has been affiliated with the *Wnt/Beta-catenin axis* (Wend et al., 2013; Wend et al. 2013). Although mutations in the *Wnt/Beta-catenin* are not so common in the HNSCCs yet other signaling cascades such NOTCH, FAT1 and AJUBA do interplay with *Wnt/Beta-catenin* to intricate changes in its activity (Beck and Golemis, 2016; Beck and Golemis 2016). Epithelial differentiation program of HPV- HNSCCs has been orchestrated by signaling cascades such as the (Ajuba LIM Protein) AJUBA, (FAT atypical cadherin 1) FAT1 and Notch drosophila homolog) NOTCH. Viral oncoproteins E6/E7 of HPV has been implemented to alter the prognosis of HPV positive HNSCCs (Liu et al., 2017). In the case of oropharyngeal squamous cell carcinoma (OPSCCs), viral oncoprotein E6 has been involved in nuclear localization of beta-catenin via activation of (Epidermal growth factor-like receptor) EGFR pathway. Inhibition of E6 with small interfering RNAs resulted in the decreased expression of the EGFR receptor activity in OPSCCs via de-mobilization of the beta-catenin into the nucleus in vitro (Nwanze et al., 2015; Hu et al., 2015). It has come to limelight less lately that certain microRNAs have certain implications in attenuation of HPV+/HPV- HNSCCs. Histone modifications and acetylation results in modulation of the *Wnt/Beta-catenin* downstream signaling. Microarray analysis conducted by the Sannigrahi et al. showed that miR-132-5p overexpression targeted *Wnt/Beta-catenin* mediated signal transducer (Sannigrahi et al., 2018). Further studies are required to elaborate on the role of the E6/E7 in the modulation of the HPV+ HNSCCs. Mesenchymal-Epithelial transition factor c-Met or Hepatocyte growth factor Receptor (HGFR) have been reported to interplay with the *Wnt/Beta-catenin* Pathway in HNSCCs (Arnold et al., 2017). Involvement of c-Met and *Wnt/ Beta-catenin* has been studied well in Colon cancer cells where their activity harness the cell fate determination in cancer stem cells. However, in HNSCCs, induction of c-Met inhibitor resulted in the elimination of CSCs in the presence of Beta-catenin. Frizzled class receptor 8 (FZD8) a modulator of *Wnt/Beta-catenin *pathway was reported to increase the expression of CSCs in HNSCCs via reactivation of (extracellular regulated MAP kinase) ERK/c-Fos signaling axis (Arnold et al., 2017). These findings suggest the fact that various cellular cascades modulate *Wnt/Beta-catenin* signaling. 

Even though modulated *Wnt *signaling has been well understood in diverse cancer types, the role of *Wnt* in the HNSCC first came to light by the study conducted by Leethanakul et al. that observed the effects of *Wnt* using microarray technology. Also involvement of (Frizzled) Fzd and (Dishevelled) DvL homologs were compared with normal tissue samples. Their findings revealed that both of these target genes were over-expressed suggesting the intrusion of *Wnt*-mediated canonical signaling in the development of HNSCCs (Leethanakul et al., 2000). To further support this hypothesis, experiments conducted by Lee et al. showed that the *Wnt* homologs (such as *Wnt1* and *Wnt10 b*) were quite common in HNSCC tissues and expressed at higher values. Conclusively, Fzd and DvL are co-related the modulated *Wnt* signaling in the development of HNSCCs (Lee et al., 2014).

More recent advances in the *Wnt*-mediated signaling is its involvement in the CSC differentiation and development. Aberrant *Wnt* signaling has been directly associated with the over-expression and differentiation of CSC. Indeed CSC survival and differentiation and *Wnt* seem strictly coupled: activated *Wnt* signaling is indispensable for cancer development (Augustin et al., 2017). 

A study conducted by Semenov et al. reported the crucial role of *Wnt* signaling in the development of breast cancer via CSC. Their findings suggest that aberrant *Wnt* signaling promotes the accumulation of several downstream targets such as Axin2 that promotes stable beta-catenin necessary for cell survival. Furthermore, these downstream targets block the efficacy of *Wnt* signaling inhibitors such as DKK1 resulting in the persistent survival of CSC. Altogether this suggests that *Wnt* signaling is differentially expressed in normal and CSC (Semenov et al., 2001).

*Wnt and Drug Resistance in HNSCC*

Disease relapse is a major hallmark in HNSCC primarily due to the presence of drug-resistant CSC. These cells foster the development of more resilient phenotypes that negate any possible way of apoptosis and thus produce more aggressive tumors (Yanamoto et al., 2014). Tones of evidence have indicated the role of *Wnt* in conferring resistance to chemotherapy by up-regulating the CSC activity in HNSCC. Warrier et al. using (secreted frizzled-related protein 4) SFRP4, an antagonist of Fzd/*Wnt*, demonstrated that SFRP4 increased drug sensitivity by 25% in HNSCCs. They showed that SFRP4 directly competed with the *Wnt* and greatly enhanced the effects of cisplatin-induced apoptosis and reduced tumor cell viability. Moreover, the antagonist did not affect the normal CS indicating the fact that *Wnt* signaling has a mighty role in the development and differentiation of CSC related to HNSCC (Warrier et al., 2014).

The mechanisms underlying the up-regulation of chemoresistance in CSC are still poorly understood and how *Wnt* mediates the activation of induced CSC is still a dilemma. However, compelling evidence has shed light on a set of transporter proteins such as the ABC family. These cell transporters have been strongly associated with an astounding chemoresistance in a broad range of tumor cell lines. Transporter proteins affect the drug efflux of the cell resulting in a massive anti-apoptotic effect(Chen et al., 2013). The most suitable candidate involved in disrupting the normal drug efflux in HNSCC is ABCG2 (ATP-binding cassette sub-family G member 2). This transporter protein seems to be the sole cause of chemoresistance as it is highly upregulated in HNSCC {Guan, 2015 #83}. Five types of ABC transporters namely ABCC1 to ABCC5 have been considered to be prime regulators in the classical activation hyper-activation of *Wnt* signaling pathway in spheroid cells of HNSCC. Furthermore, the ability of spheroid cells to produce chemoresistance triggered by CSC was completely exhausted by knocking out the genes responsible for beta-catenin synthesis. This knock-out also contributed to the loss of SC markers necessary for self-renewal (Song et al., 2010; Yao et al., 2013). Despite these evidence the actual cross talk that underlies drug resistance regulated by the interplay between *Wnt* Pathway and ATP binding cassette family (ABC) proteins still needs to be explained. However, further developments in unraveling the interaction between *Wnt* and ABC proteins will show the potential of how ABC transporter Protein channel regulates the overexpression of CSCs in HNSCCs (Chikazawa et al., 2010).

*Wnt and Druggable Targets*

In recent years, preclinical trials have determined a broad class of synthetic as well as natural compounds that have been implicated in *Wnt* signaling dysregulation in cancer (Lee et al., 2011; Mahtaj et al., 2015). Some of these tiny molecules have been observed to restrain *Wnt* signaling cascade at diverse steps including activation of target proteins and modulation of the enzymatic activity responsible for *Wnt*-mediated cell growth. Thus, they hold promising benefits in devising strategies to understand *Wnt* signaling. It is the need of the hour to explore the potential of these promising biosynthetic molecules for promising therapy against *Wnt*-mediated cancer (Verkaar and Zaman, 2011). 

To suppress *Wnt* signaling, a variety of approaches have been utilized: one of them is a blockade of *Wnt* activity by definite inhibitors. PORCN also was known as a porcupine is an enzyme mainly restrict the activation of *Wnt* signaling at serine residues. PORCN activity promotes the palmitoylation of the *Wnt* (Proffitt et al., 2013). Proffitt et al. demonstrated the sudden decrease in the expression of *Wnt* signaling by using small inhibitors of PORCN such as* ETC-159* and *Wnt-C59*. Their findings shed light on how PORCN deficiency mediates the interplay between *Wnt* and Fzd proteins. PORCN directly stalls the production of excessive *Wnt* and consequently inhibits the interaction between *Wnt* and *Fzd* proteins. Therefore *Wnt* downstream signaling is inhibited, and cancer growth can be potentially hindered. The efficacy of *Wnt* inhibition by PORCN has been established in various animal models and cell lines (Liu et al., 2013; Proffitt et al., 2013; Madan et al., 2016).

Furthermore, Liu et al., (2013) evidenced the synergy between inhibitors of PORCN such as LG974 and *Wnt* signaling in the rodent model of breast cancer. The effectiveness of these small molecules as inhibitors of *Wnt* has been established in almost all formats of cancer in various mammalian models. Moreover, PORCN inhibitors have been demonstrated to play a pivotal role in the down-regulation of *Wnt* target genes with minimal toxicity to the normal tissues.

In *Wnt*-mediated mice tumors of mammary glands, Kabiri et al., (2015) demonstrated that using *Wnt-C59 *(*Wnt* inhibitor) had no significant effect on the normal blood cell count suggesting the fact these inhibitors can block “cancerous” *Wnt* signaling with precision and have almost zero toxicity in normal hematopoietic SC. Furthermore, the authors speculated that other possible mechanisms for self-renewal of normal SC than the abrogated *Wnt* signaling could be present. They also suggested that there might be a competition between lower levels of *Wnt* and the adaptability of SC and this could force SC to proliferate using a different set of mechanisms. Interestingly, compelling evidences have suggested the involvement of these inhibitors in tumor regression having little or almost no side effects.

In seeking for *Wnt* signaling biosynthetic inhibitor, Huang et al. demonstrated that XAV939 has greater efficacy installing *Wnt* signaling by targeting Tankyrases (TNKS). TNKS have been reported to be essential mediators for the synthesis of Axin. Indeed inhibition of Axin promotes degradation of beta-catenin resulting in the delayed or no activation of *Wnt* signaling (Trosset et al., 2006; Huang et al., 2009).

Other pharmacological approaches for limiting *Wnt* signaling involve protein-protein interactions. Disrupting the interaction between proteins can help to promote the down-regulation of *Wnt* signaling. PNU74654 has been considered a promising drug for *Wnt*-mediated tumors (Dihlmann et al., 2003). It has the potential to block the interplay between the transcription of T-cell receptor factor 4 (TCF4) and beta-catenin. It inhibits their interaction resulting in blocking of the *Wnt* signaling (Dihlmann et al., 2003; Bos et al., 2006). Acetylsalicylic acid (ASA) and other anti-inflammatory and antipyretic drugs have been implicated in *Wnt* signaling inhibition in various cancers. Dihlmann et al., (2003) demonstrated the involvement of ASA in mediating initiation of phosphorylation at serine residues of beta-catenin. This promotes inhibition of *Wnt* signaling in the colorectal carcinoma. Furthermore, ASA accelerates the phosphorylation at Tyr307 in PP2A enzyme a member of Heat repeat protein family. This phosphorylation promotes the interaction and phosphorylation of the beta-catenin, resulting in the suppression of the canonical *Wnt* signaling (Gao et al., 2009; Leow et al., 2010). 

Among natural inhibitors of *Wnt* signaling, curcumin has been investigated extensively. It plays a vital role in the suppression of the *Wnt* canonical signaling, through a dose-dependent manner (Aminuddin and Ng, 2016). Curcumin inhibits the nuclear localization of beta-catenin and also blocks the interaction between *Wnt* and TCF4. Furthermore, other natural compounds as extracts from green tea have been implicated in lung cancer growth inhibition. Extracts directly interact and promote the unmethylation of early methylated genes such as WIF1. WIF1 is an antagonist of *Wnt* signaling and thus can inhibit the downstream signaling of the *Wnt* signaling (Lee et al., 2011; Smith et al., 2013; Yeung et al., 2014; Fischer et al., 2017; Nguyen et al., 2009) 1,8-cineol a terpenoid is another biologically active natural compound that has been reported to inhibit *Wnt/Beta-catenin* signaling cascade in HNSCCs via targeting GSK-3 an essential regulatory protein of the *Wnt* pathway. Glycogen synthetase Kinase 3 (GSK-3) interacts with APC that in turn destroys the active Beta-catenin complex. Using HNSCCs Cell lines Roettger et al. demonstrated that 1,8-cineol was responsible for reducing *WNT11* mediated cellular proliferation in HNSCCs (Roettger et al., 2017). Despite these powerful improvements in the search for *Wnt* signaling modulators, fewer drugs have been entered in the clinical use CSCs is one stumbling block that has hampered suitable drug formulation for HNSCCs. Diverse cell-population, high rate of metastasis, migration and invasion properties of the CSCs might be the major setbacks in development of an effective drug for the *Wnt*-mediated HNSCCs. Tumor recurrence is another hindrance in the development of a drug. Intrinsic factors such as the transmembrane proteins, incubation stages and mutations in the apoptotic machinery are also chief culprits responsible for the resistance to cytotoxic agents and radiation. Another dilemma for the development of effective drug therapy is the self-renewal capacity of the cancer stem cells. Radiation therapy has little or no influence on the CSCs as they can produce new stem cells instantly (Yeh et al., 2016). OMP-54F28, a chimeric product of human IgG1 and frizzled protein Fzd8 have been implicated in reducing growth in pancreatic cancer cells. Interestingly, this drug has been entered in different clinical trials, but results are still to be reported. Only one work, a phase I clinical trials (Jimeno et al., 2017), underlined as OMP-54F28 was well tolerated in patients with solid tumors. Indubitably more research is needed to address the role of *Wnt* signaling modulators in cancer.

In conclusion, a massive improvement is still required in developing an effective therapeutic intervention for targeting *Wnt* signaling in tumors. Different preclinical trials have opened new horizons, searching for new therapeutic interventions are still required for *Wnt*-mediated cancer. Furthermore, strategies and techniques are required to cope with CSC produced through aberrant *Wnt*-mediated signaling. As these cells share the same characteristics (self-renewal, differentiation) as the normal human SC, it is necessary to devise methodologies that will be more effective and precise in treating such CSC. *Wnt* signaling is the main mediator for the production of CSC concerning HNSCC. Evaluating the various aspects of signaling can diversify insight into this crucial pathway and will help in understanding the cross-talk between different cellular signaling pathways. This will enable to develop broad therapeutic interventions for both eradicating as well as coping with the disease relapse in HNSCCs. 

Cancer is a multifactorial disease and targeting a specific pathway in such a complex pathology is hindered by several obstacles. Toxicity, allergic reaction, immune suppression, and chemo-resistance, non-specific off-target binding are some of the several factors that impede the tailored treatment of cancer. Overcoming such hurdles can enhance the chances of therapy to maximum potential. Moreover, improving drug development and efficacy by choosing the right target or using a combination of drugs can narrow the possible side effects and guarantee the best therapeutic value. Complex and profound studies on the efficacy, safety, and toxicity of candidate drugs are fundamental to discover novel agents that potentially have low side effects and great medical benefits overcoming health risks for cancer patients.

## Conflicts of Interest

The authors declare no conflict of interest.
